# Role of Vitamin D in Prevention of Food Allergy in Infants

**DOI:** 10.3389/fped.2020.00447

**Published:** 2020-08-18

**Authors:** Arianna Giannetti, Luca Bernardini, Jessica Cangemi, Marcella Gallucci, Riccardo Masetti, Giampaolo Ricci

**Affiliations:** Department of Paediatrics, S. Orsola-Malpighi Hospital, University of Bologna, Bologna, Italy

**Keywords:** vitamin D, food allergy, prevention, allergic sensitization, immune system

## Abstract

The prevalence of food allergy is increasing over the last decades. The role of vitamin D in the prevention of food allergy has been largely investigated. Its role on the physiology of calcium and bone is known, but calcitriol (active form of the vitamin D) also influences the epithelial cells, T cells, B cells, macrophages, and dendritic cells. Almost all cells of the adaptive immune system express the vitamin D receptor, making them also capable of being vitamin responsive. Specifically considering the potential role of vitamins in food allergy, vitamin D has been shown to affect several mechanisms that promote immunologic tolerance, including the T regulatory cell function and the induction of tolerogenic dendritic cells. The target of our review is to evaluate the role of vitamin D in the prevention of food allergy in children. There are contradictory data on the relationship among the vitamin D deficiency and the developing of food allergy. Some studies associate lower exposure to sunlight to food allergy; on the other hand, further research has found that higher vitamin D levels could increase the likelihood of allergic sensitization and food allergy. Therefore, there is an urgent need for well-planned randomized controlled trials on vitamin D supplementation, with particular regard to the prevention of food allergy. The role of vitamin D beyond bone and calcium metabolism is not fully understood.

## KEY POINTS

- Vitamin D is a hormone with pleiotropic effects, essential not only for calcium homeostasis and bone mineralization but also for the proper functioning of the immune system.
- However, some patients do not benefit from vitamin D supplementation owing to genetic alterations in metabolism rather than absorption.
- The association between vitamin D and development of food allergy is contradictory.
- There is a potential association between lower sunlight exposure and food allergy, but on the other side, it appears that higher levels of vitamin D might raise the probability of allergic sensitization and food allergy.
- Vitamin D must be considered as a further chance in comprehension and treatment of atopic diseases.
- There is an urgent need for well-planned randomized controlled trials on vitamin D supplementation in food allergy.

## Introduction

**Figure 1:**
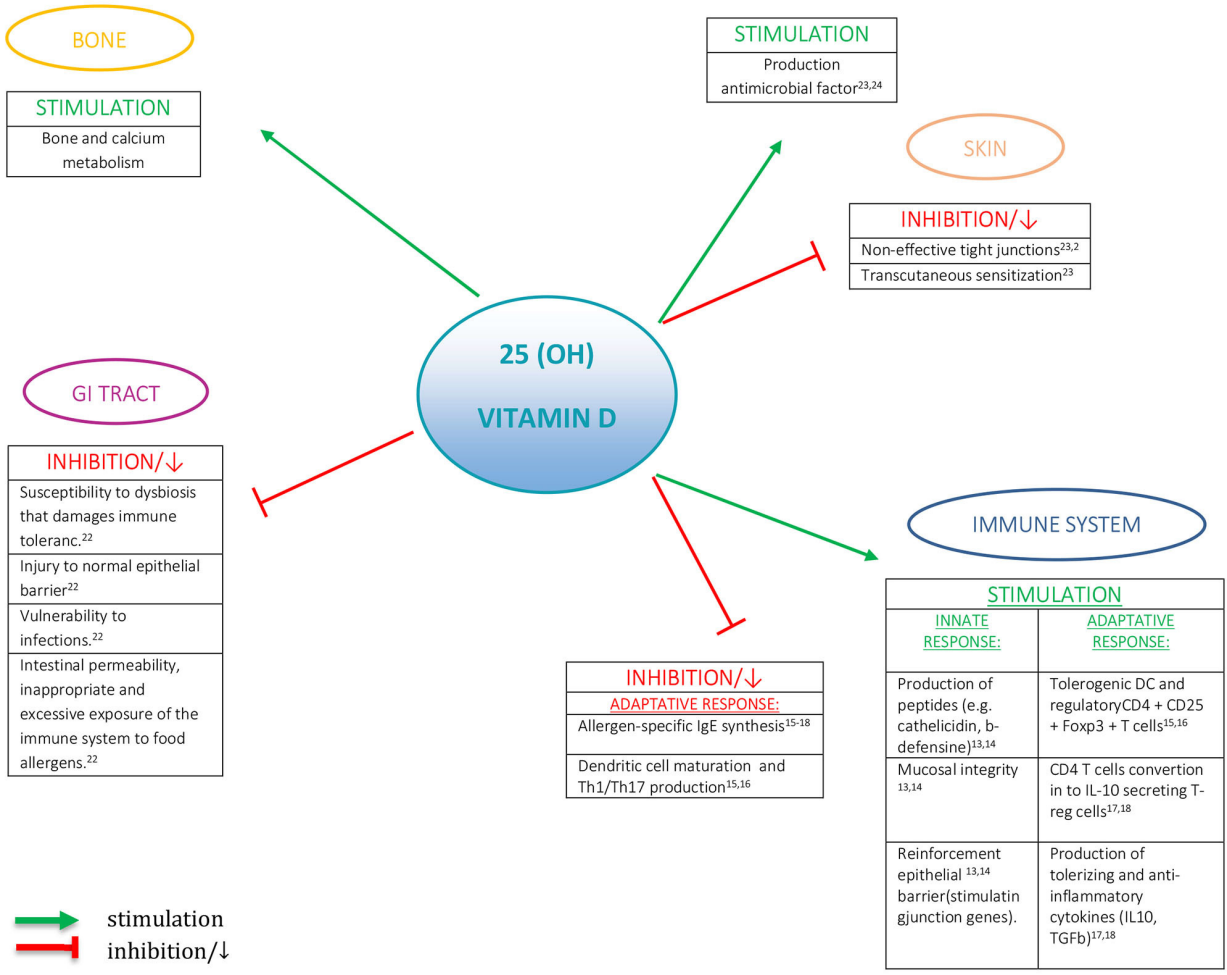
The role of vitamin D.

In recent decades, the occurrence of food allergies has recorded a significant increase in many developed countries worldwide, probably as a result of environmental and lifestyle changes (1, 2). Vitamin D is a hormone with pleiotropic effects, essential not only for calcium homeostasis and bone mineralization but also for the regulatory effects on the immune system (Figure 1) (3).

Vitamin D receptors (VDRs) are expressed in almost all the tissues of the human body. There is a significant association between vitamin D levels and the risk of immunologic, metabolic, or neoplastic disorders (4). Moreover, epidemiological evidence suggests a role of vitamin D in food allergy pathogenesis (5). Vitamin D impacts the function of macrophages, dendritic cells, B cells, T cells, and epithelial cells, playing a key role in immune response, both innate and adaptive (6, 7). All these cells convert the circulating prohormone form into the active one. Thanks to vitamin D, the innate response is capable to express antimicrobial peptides—such as cathelicidin—and plays an important role for keeping mucosal integrity and reinforce epithelial barrier by stimulating junction genes (8, 9). Regarding the adaptive immune response, it was found that VDR agonists affect the function of Th1 and Th2 cells, stimulate tolerogenic dendritic cells and regulatory CD4^+^CD25^+^ Foxp3^+^ T cells, avoid dendritic cell maturation, and abolish allergen-specific IgE synthesis (10, 11). In *in vitro* studies, by exposing human CD4 T cells to 1,25(OH)_2_D, their conversion into IL-10 secreting Treg cells has been proven; at the same time, there is a suppression of IgE making by B cells, with production of antiphlogistic and tolerogenic cytokines (12, 13).

In our study, we reviewed the current literature to evaluate the role of vitamin D in food allergy in children.

## Vitamin D Physiology

In humans, the major source of vitamin D (90%) is the exposure to solar UVB radiation (290–315 nm wavelengths), which determines the formation of cholecalciferol in the skin, which is then metabolized in the liver to 25-hydroxyvitamin D (25-OH-D_3_) and finally carried to the kidneys, where it is transformed into the active form [1,25-dihydroxyvitamin D, 1,25-(OH)_2_D] (3, 14). Only 10% of vitamin D is obtained through food ingestion.

The best indicator of vitamin D status is serum 25(OH)D_3_ levels, which reflect the whole intake of vitamin D, comprehensive sun exposure, integrations, and food intake. In recent decades, it has been observed that there is an increasing evidence of a global vitamin D deficiency (VDD) for all ages (15, 16) owing to a combination of extrinsic and intrinsic factors. In the first group, we can include the intensity of exposure to UVB determined by seasons, latitude, altered eating habits, and behavioral factors, without forgetting campaigns to prevent skin cancer, whereas in the latter we might mention the individual level of skin melanin content and the intestinal absorption of vitamin D. Obese people have a greater risk of VDD, caused by their lifestyle and also probably as a consequence of the uptake in the adipose tissue of this liposoluble vitamin (17). However, some patients do not benefit from vitamin D supplementation owing to genetic alterations in metabolism rather than absorption. In a recent literature review, 35 genes putatively associated with abnormal serum 25 (OH)D_3_ were identified (18).

## Potential Role of Vitamin D in the Development of Food Allergy

### Hypothesis on Pathogenic Mechanisms

The cellular and molecular mechanisms involved in the pathogenesis of food allergy are very complex and encompass genetic, epigenetic, and environmental factors (19–21). Several mechanisms have been proposed aimed at clarifying the role played by VDD in the food allergy pathogenesis. VDD, at a particular time of life, might increase the susceptibility to colonization by abnormal intestinal microbial flora, contributing to increased intestinal permeability, leading to an inappropriate and excessive exposure of the immune system to food allergens. On the other hand, VDD might cause a disequilibrium at the intestinal level that damages immune tolerance, destroys the normal epithelial barrier, and increases the susceptibility to infections (22). Food allergen sensitization can also be driven by percutaneous sensitization, which may be important particularly in children with VDD (23). It can be speculated that decreased antimicrobial factor and non-effective tight junctions caused by VDD may determine, in the skin, an anomalous exposition and thereby a boost of the immune system, driving to allergic sensitization eczema (24) and the onset of food allergy (23), in addition to an important increase in the severity of atopic dermatitis (25).

The current studies in the literature on the possible role of vitamin D in the development of food allergy have been reported in Tables 1, 2, which are detailed below.

**Table 1:** Summary of studies on the possible role of vitamin D in the development of food allergy.

**References**	**Study**	**Age, sample**	**Results**	**Definition of vitamin D deficiency**
Sharief et al. (26)	Retrospective study	3,136 children/adolescents and 3,454 adults	25(OH)D levels <15 ng/ml associated with peanut allergy, no consistent associations seen in adults	25(OH)D deficiency <15 ng/ml, insufficiency 15–29 ng/ml
Mullins et al. (27)	Retrospective study	115 peanut allergic patients younger than 72 months	Non-linear relationship between neonatal 25(OH)D_3_ levels and peanut allergy in children under 6 months of age, slightly higher levels (75–99.9 nmol/L) linked with lower vs. those in the reference group (50–74.9 nmol/L)	Neonatal concentration of 25(OH)D divided into four groups: <50, 50–74.9, 75–99.9, and >100 nmol/L. The reference group was considered between 50 and 74.9 nmol/L
Kim et al. (28)	Retrospective study	18,181 patients 10 years or older (2,814 patients with food-induced anaphylaxis and 15,367 people with available serum vitamin D measurements)	Higher incidence of food-induced anaphylaxis in regions with lower vitamin D levels in the population	Not defined
Kull et al. (29)	Prospective birth cohort	4,089 newborn infants were followed for 4 years	Water-soluble form increased the risk of allergic disease in children up to the age of 4 years compared with supplementation of same vitamin given in peanut oil	Not defined
Camargo et al. (30)	Prospective pre-birth cohort study	1,194 mother–child pairs followed up through age 3 years	Higher maternal intake of vitamin D during pregnancy may decrease the risk of recurrent wheeze in early childhood	Not defined
Nwaru et al. (31)	Prospective cohort study	971 children with 5-year follow-up	It was found that maternal intake of vitamin D was inversely associated with sensitization to food allergens	Not defined
Liu et al. (32)	Prospective birth cohort study	649 children who were enrolled at birth and followed from birth onward	Vitamin D deficiency may increase the risk of food sensitization among individuals with certain genotypes	Cord blood 25(OH)D_3_ <11 ng/ml
Jones et al. (33)	Prospective birth cohort study	231 mother–child pairs, derived from a larger (*n* = 669) prospective birth cohort, followed up until 1 year of age	Reduced fetal exposure to vitamin D increases the risk of eczema in infants by 12 months of age	25(OH)D_3_ levels cutoffs were divided in <50 nmol/L, 50–74.99 nmol/L, >75 nmol/L
Weisse et al. (34)	Prospective	378 mother–child pairs followed up until 2 years of age	High vitamin D levels in pregnancy and at birth may contribute to a higher risk for food allergy	The assay detection limit was defined as 6.7 ng/ml for maternal 25(OH)D_3_ and 5.2 ng/ml for maternal 25(OH)D_2_. Detection limit for cord blood 25(OH)D_3_ and D_2_ was 3 ng/ml
Allen et al. (35)	Australian large prospective cohort study	577 infants, 1 year of age	Vitamin D insufficiency more likely associated with peanut and/or egg allergy. Vitamin D insufficiency linked to multiple food allergies (≥2) rather than a single food allergy	Vitamin D insufficiency: ≤ 50 nmol/L
Chiu et al. (36)	Prospective study	186 children (0–4 years)	Cord blood 25(OH)D levels inversely linked with the risk of milk sensitization at 2 years of age	Low vitamin D levels <20 ng/ml
Chawes et al. (37)	Prospective clinical study	257 children	Cord blood 25(OH) vitamin D levels defined as <50 nmol/L was not associated with allergic sensitization	Cord blood 25(OH)-Vitamin D: deficient, 50 nmol/L; insufficient, 50–75 nmol/L; sufficient, >75 nmol/L
Hennessy et al. (38)	Prospective Cork BASELINE Birth Cohort Study	Vitamin D was measured in maternal sera at 15 weeks of gestation (*n* = 1,537) and umbilical cord blood (*n* = 1,050)	The investigators did not observe any association between vitamin D during pregnancy or at birth with allergic disease outcomes at 2 and 5 years old	Maternal 25 (OH) D divided into <30 nmol/L; 30–49.9 nmol/L; 50–74.9 nmol/L; ≥75 nmol/L
Ercan et al. (39)	Prospective, observational, case–control study	111 children <2 years of age	No statistically significant relationship between the CMPA group and healthy controls in terms of 25(OH)D levels	Vitamin D deficiency ≤ 20 ng/ml, insufficiency 21–29 ng/ml, adequate ≥30 ng/ml
Sardecka et al. (48)	Prospective two-stage study	138 infants with CMA and 101 healthy infants	Children with increased Foxp3mRNA expression (predictive of faster gain of tolerance in infants with CMA) have lower serum vitamin D levels than healthy children	25 (OH)D concentration sufficient ≥30 ng/ml for the Polish population
Baek et al. (49)	Cross-sectional study	226 children aged 3–24 months with atopic dermatitis or suspected food allergy	VDD increased the risk of food allergen sensitization especially to milk and wheat. The polysensitization group had significantly lower levels of 25(OH)D than the non-sensitization and monosensitization group	Serum 25(OH)D levels: deficiency, <20.0 ng/ml; insufficiency, 20.0–29.0 ng/ml; and sufficiency, ≥30.0 ng/ml
Rosendahl et al. (50)	Randomized controlled study	975 infants followed up until 12 months of age	No differences between the vitamin D supplementation groups in food sensitization at 12 months. Possible adverse effect of high concentrations of vitamin D	25 (OH)D_2_ considered sufficient for concentrations ≥50 nmol/L
Guo et al. (51)	Large observational study	2,642 children followed up until 2 years of age	No evidence found supporting the link between low levels of 25 (OH)D and allergic sensitization to various allergens	25(OH)D concentrations insufficient <75 nmol/L and sufficient otherwise
Thorisdottir et al. (52)	Longitudinal Icelandic study	144 children followed up until 6 years of age	At 12 months, IgE-sensitized children had a lower intake of vitamin D, but no significant difference in mean serum 25(OH)D was found between IgE-sensitized and non-sensitized children, nor at 12 months or 6 years	Vitamin D deficient: <30 nmol/L and vitamin D intake from diet and supplements combined did not exceed 25 μg/day in infancy or at 6 years

**Table 2:** Summary of review on the possible role of vitamin D in the development of food allergy.

**References**	**Study**	**Age, sample**	**Results**	**Definition of vitamin D deficiency**
De-Regil et al. (53)	Cochrane review	Variable	Vitamin D supplementation, during pregnancy, both single-dose or continued, increased 25(OH)D_3_ levels at term; however, the clinical implication of improving the vitamin D concentration and the possible use of this intervention strategy as part of the routine antenatal care are yet to be evaluated	Variable
Mirzakhani et al. (54)	Review	Variable	Well-designed and well-powered clinical trials are needed to determine whether supplementation of vitamin D should be recommended in allergic diseases	Variable
Willits et al. (55)	Review	Variable	No association between food allergy and vitamin D level	Variable
Yepes-Nuñez et al. (56)	Systematic review of randomized and non-randomized studies	Variable	Vitamin D supplementation for pregnant women, breastfeeding women, and infants may not decrease the risk of developing allergic diseases, such as atopic dermatitis (in pregnant women), allergic rhinitis (in pregnant women and infants), asthma and/or wheezing (in pregnant women, breastfeeding women, and infants), or food allergies (in pregnant women). However, they conclude that the potential impact of vitamin D on food allergy remains uncertain	Variable
Saggese et al. (57)	Review	Variable	In food allergies, the role of vitamin D remains controversial	Variable
Hawrylowicz et al. (58)	Review	Variable	Longitudinal studies of vitamin D requirements *in utero* and post-natally, better understanding of factors that influence bioavailability of vitamin D, and mechanistic insights into vitamin D effects on neonatal-specific immune pathways are awaited	Variable
Matsui et al. (59)	Review	Variable	Fall and winter birth could worse food sensitization	Variable

### Evidence on VDD and Anaphylaxis

The first reports regarding a possible association between food allergy and VDD came from the observation that there was a direct relationship among increasing latitude and cases of anaphylaxis, prescription of epinephrine autoinjector, or food allergy–related admissions (30, 40–44). Contrasting results have been reported about the correlation between vitamin D status and atopic dermatitis severity (45). A recent Korean study (28) compared incidence of food-induced anaphylaxis (FIA) and vitamin D serum levels between two regions of high and low solar radiation, finding that, in the region of lower solar radiation, vitamin D levels were lower, with concomitantly higher FIA incidence. These findings suggested the possible causal function of vitamin D levels in food allergy, but data sources of FIA and vitamin D used in the study differed (27, 28, 30, 40–44, 46). Kim et al. (28) designed a study that included 2,814 patients with FIA and 15,367 people with available serum vitamin D measurements. After stratification by age, sex, and area of residence, lower solar radiation region had higher FIA incidence (2.2 per 100,000 person-years vs. 1.8 per 100,000 person-years) and lower vitamin D values (16.5 vs. 17.8 ng/ml) than higher solar radiation region. Camargo et al. (42) examined regional rates of epinephrine autoinjector (EpiPen) prescription in the United States, finding a strong north–south gradient. Mullins et al. (44) evaluated epinephrine autoinjector prescriptions and anaphylaxis hospital admission rates in Australia, used as surrogate markers of anaphylaxis. Both in an unadjusted and adjusted model of children from birth to the age of 4 years, they found a decrease in EpiPen prescription as decreasing absolute latitude. The anaphylaxis admission rates also showed a similar gradient. These data provided additional support and etiologic clues for a possible role of vitamin D in anaphylaxis pathogenesis. However, we cannot demonstrate that food allergy is linked to vitamin D levels and not to any other geographic, seasonal, or sunlight-derived factor (47).

### Evidence on VDD and Season of Birth

Other studies showed a relationship between less sunny season of birth and increase of food allergy prevalence. Seasonal differences in UVB exposition result in reduced 25(OH)D_3_ levels in autumn and winter months; in the higher latitudes, there is no sufficient UVB intensity in the cooler months for proper synthesis of 25(OH)D_3_ to occur, irrespective of sunlight exposure (27). In addition, there are various data that hypothesize the possible link between season of birth and food allergy. The potential mechanisms are consequential to VDD owing to a paucity of UVB exposure. Matsui et al. (59), in their recent review, proposed the hypothesis that autumn and winter birth could worsen eczema, with the risk of excessive food antigen exposure and sensitization. Moreover, the deficiency of UVB exposure may lead to inadequate Treg expansion, potentially responsible for impaired food tolerance regardless of VDD. VDD resulting from an inadequate vitamin D synthesis from skin could compromise the intestinal epithelial barrier and antimicrobial peptides, with the risk of intestinal dysbiosis. Lastly, VDD could also modulate immune response and lead to sensitization and impaired food tolerance. Limitations of considered studies are a precise definition of vitamin D deficiency and the presence of co-factors in the population, such as eczema, skin color, race, residence, skin color, gender, and age. The apparent immune suppressive effect of ultraviolet radiation is not limited to the supposed inverse relationship between vitamin D levels and FIA rate, but it is also thought to be involved in the development of immune-related disorders, such as type 1 diabetes mellitus (43).

### Evidence on VDD and Allergic Sensitization

The milestone NHANES study (26), based on extensive nationally representative samples from the United States of more than 3,000 children and adolescents, found an association between VDD and higher levels of specific IgE, and thereby allergic sensitization to several allergens, both environmental and food, in children and adolescents, but not in adults. At the same time, the relationship between increased IgE and excessively high vitamin D levels was not confirmed. By contrast, Hypponnen et al. (60), in an adult population study, showed that both low and excessive circulating 25(OH)D_3_ levels were correlated with an increase in IgE in a non-linear relationship. Furthermore, higher rates of food sensitization have been seen in infants born to mothers with low vitamin D intake in pregnancy. Nwaru et al. (31) examined the effect of maternal diet during pregnancy on allergic sensitization in a population-based cohort study with 5-years follow-up, by evaluating 971 children with human leukocyte antigen–caused predisposition to type 1 diabetes, for whom maternal pregnancy dietary survey and allergen-specific IgE measurements at 5 years were recorded. The data showed an inverse correlation between sensitization to food allergens and maternal intake of vitamin D, whereas intake of citrus fruits in childbearing might raise the risk of developing allergic sensitization in the sons. A Korean cross-sectional study (49), which included 226 infants [168 infants with atopic dermatitis (74.3%) and 58 with suspected food allergy without atopic dermatitis (25.7%), aged 3–24 months], demonstrated that VDD increased the risk of food allergen sensitization, especially to milk and wheat, but in this work, the diagnosis of food allergy was only suspected and not confirmed by oral food challenge. An Australian large prospective cohort study proved that infants with low vitamin D levels (25(OH)D_3_ <50 nmol/L) at 12 months of age were more likely to be affected by challenge-proven food allergy, in particular to peanuts and egg, and to have multiple food allergies, in comparison with those who had appropriate vitamin D levels. Curiously, this connection was clear only among infants of Australian-born parents, hinting a gene–environment interaction (35). However, other studies did not support this evidence (55). Kull et al., in a prospective birth cohort of 4,089 infants, showed that vitamin D in water-soluble form increased the risk of allergic disease in children up to the age of 4 years, compared with supplementation of same vitamin given in peanut oil, but vitamin D levels were not measured at baseline nor follow-up (29).

In a prospective, observational, case–control study that involved 111 children <2 years of age, Ercan et al. (39) assessed the possible link between cow's milk protein allergy (CMPA) and 25(OH)D_3_ levels in infants with an initial diagnosis of CMPA. Moreover, they also evaluated the association between 25(OH)D_3_ levels and skin prick test induration size, specific IgE to milk and specific IgE to casein. For the study purpose, they considered vitamin D deficiency if 25(OH)D_3_ values were ≤ 20 ng/ml, insufficiency if they were between 21 and 29 ng/ml and adequate when they were ≥30 ng/ml. No statistically significant relationship was found between the CMPA group and healthy controls in terms of 25(OH)D_3_ levels, nor even milk antigen induration diameter and vitamin D levels of CMPA infants. Hence, they concluded that, at the starting diagnosis of infants with CMPA, routine workup of vitamin D levels may have no benefit. Sardecka et al. (48) examined the relationship between Foxp3mRNA expression (the best marker for Treg lymphocytes) and serum concentration of vitamins D and C, and the development of different phenotypes of tolerance in children with CMPA. The results suggest that increased of Foxp3mRNA expression can predict faster tolerance acquisition in infants with CMA. Regardless of whether they acquire tolerance, children with CMPA have lower serum vitamin D levels than healthy children. Recently, also Guo et al. (51) performed a large observational study involving 2,642 children with the aim of evaluating the correlation between serum 25(OH)D_3_ and allergic sensitization among childhood 0–2 years of age. Vitamin D was considered insufficient when serum concentration of 25(OH)D_3_ was <75 nmol/L and sufficient otherwise. They did not find evidence supporting the link between low levels of 25(OH)D_3_ and allergic sensitization to various allergens.

### Evidence on VDD, Pre-natal Data, and Birth Cohort Studies

Another matter investigated by some authors has also been the potential link between 25(OH)D_3_ concentration in newborns as a marker of risk of future development of food allergies. In the study of Mullins et al. (61) on 115 patients younger than 72 months, it was found that there is an association between peanut allergy and neonatal 25(OH)D_3_ levels. In comparison with the reference group (50–74.9 nmol/L), 25(OH)D_3_ levels of 75 to 99.9 nmol/L were linked to a reduced risk of peanut allergy. At levels of 100 nmol/L or higher, no additional reduction was found, whereas the probability of peanut allergy at levels lower than 50 nmol/L was substantially equal to that of the reference group. The risk of peanut allergy at levels <50 nmol/L was also not significantly different from the reference group. Jones et al. (33) studied the association among cord blood 25(OH)D_3_ and allergic sensitization, eczema, and food allergy at 1 year of age. The lower vitamin D levels at birth were associated to higher likelihood of eczema at 12 months, without significant differences between IgE-mediated and non–IgE-mediated eczema. Although in this high-risk subset there was a greater likelihood of IgE-mediated food allergy and allergen sensitization after the first year of life, the probability to develop IgE-mediated food allergy was not linked to cord blood 25(OH)D_3_ (33, 37). Chawes et al. (37), in their Copenhagen Prospective Studies on Asthma in Childhood (COPSAC2000) at-risk mother–child cohort, analyzed the relationship between cord blood 25(OH)vitamin D and asthma and allergy-related conditions during pre-school age in 257 children. After adjusting for season of birth, deficient cord blood 25(OH) vitamin D levels, defined as <50 nmol/L, were not associated with allergic sensitization. On the other hand, Chiu et al. (36), considering a birth cohort of Taiwanese children, found an inverse relationship between cord blood 25(OH)D_3_ levels and milk sensitization at the age of 2 years. One hundred eighty-six children aged 0 through 4 years were enrolled and regularly followed up for 4 years. The average cord blood 25(OH)D_3_ level was 23.8 ± 9.5 ng/ml, with a high occurrence of VDD (<20 ng/ml) at birth (42%). A trend was found between low cord blood 25(OH)D_3_ levels and higher risk of milk sensitization throughout childhood. At the same time, cord blood 25(OH)D_3_ levels showed an inverse relationship to the risk of milk sensitization at 2 years old, an age at which a greater occurrence of milk sensitization was markedly associated to the risk of asthma development and allergic rhinitis at the age of 4 years. Nonetheless, low cord blood vitamin D levels do not seem linked to a higher risk of allergic rhinitis, eczema, or asthma in early childhood. Hennessy et al. (38), in their Cork BASELINE Birth Cohort Study, investigated associations between intrauterine vitamin D status and atopic outcomes in an extensively characterized, disease-specific, maternal-infant cohort. In this study, the diagnosis of food allergy was made for all children during the 24-months clinical evaluation visit using skin prick tests. The panel of food allergens included cow's milk, eggs, peanuts, cod, soybeans, and wheat. In the case of wheals with a diameter of ≥3 mm, a blinded oral food challenge was completed, if the food had not been eaten previously or if there was a story that suggested the risk of food allergy. The investigators did not observe any association between vitamin D during pregnancy or at birth (measured in maternal sera at 15 weeks of gestation and umbilical cord blood) with allergic disease outcomes (eczema, food allergy, asthma, allergic rhinitis) at 2 and 5 years old. A German study (34) focused on the effects of newborn and maternal vitamin D levels and their influence on the development of food allergy in children, and considered 378 mother–child pairs during pregnancy and at childbirth atopic manifestations during the first 2 years of life by using questionnaires filled out by the parents during pregnancy and annually thereafter. They demonstrated that high vitamin D levels in pregnancy and at birth might lead to a greater risk of food allergy, suggesting that, to prevent atopy, integration is not required. Recently, Rosendahl et al. (50) realized a randomized controlled trial of daily vitamin D supplementation of 10 or 30 μg from the age of 2 weeks, measuring food and aeroallergen IgE antibodies at 12 months of age. It was demonstrated that high-dose vitamin D supplementation did not prevent allergic sensitization and allergic diseases during the first year of life. On the other hand, it was observed that there is an increased risk of milk allergy in infants randomized to the higher vitamin D supplementation and an increased risk of milk allergy in infants with high cord blood vitamin D status, thereby suggesting a possible adverse effect of high concentrations of vitamin D. A Cochrane review (53) proved that vitamin D supplementation, during pregnancy, both single-dose or continued, increased 25(OH)D_3_ levels at term; however, the clinical implication of enhancing the vitamin D concentration and the possible role of this approach in the standard antenatal care are still to be evaluated because of the limited number of trials and outcomes to deduce implications on safety and efficacy. The presence of mixed results may be a consequence of the gaps in our knowledge about the precise role of vitamin D in the development of food allergy (47). Yepes-Nuñez et al. (56), in a recent systematic review including randomized and non-randomized studies, showed that vitamin D integration for pregnant women, breastfeeding women, and infants might not reduce the probability of allergic disease development, such as food allergies (in pregnant women), asthma and/or wheezing (in pregnant women, breastfeeding women, and infants), allergic rhinitis (in pregnant women and infants), or atopic dermatitis (in pregnant women). However, they concluded that the potential impact of vitamin D on food allergy remains uncertain. Liu et al. (32) evaluated the association among cord and maternal vitamin D level (VDD if cord blood 25(OH)D_3_ <11 ng/ml) and atopic outcomes in 649 children recruited at birth and followed from then on, and found that VDD alone was not related with food sensitization. If examined together with SNPs, a significant interaction was found between VDD and IL-4 gene polymorphism. VDD raised the risk of food sensitization among children carrying CC/CT genotypes; comparable but lower relationships were seen for the SNPs. The conclusion of their study is that VDD may enhance the risk of food sensitization among people with specific genotypes. A recent longitudinal Icelandic study, involving 144 children followed up for 6 years, compared infant feeding with particular regard to vitamin D supplementation and 25(OH)_3_ levels between IgE-sensitized and non-sensitized children at 6 years. They found that, at 1 year of age, IgE-sensitized children had a reduced intake of vitamin D, partially explained by a reduced, but non-significant, vitamin D supplement use and reduced consumption of vitamin D fortified formula. At 6 years, less IgE-sensitized children used vitamin D supplements regularly. Equally, vitamin D integration at 6 years decreased the ratio of IgE sensitization (52). Nonetheless, the authors did not record a difference in mean serum 25(OH)D_3_ between IgE-sensitized and non-sensitized children, nor at 12 months (96.8 ± 33.6 vs. 99.3 ± 32.2 nmol/L, respectively) or 6 years (59.3 ± 15.9 vs. 56.0 ± 16.7 nmol/L, respectively). In conclusion, their data encouraged, for Nordic infants and children, vitamin D intake from diet and supplements.

Further issues are about the definition of optimum, deficiency, and insufficiency vitamin D serum levels, not worldwide recognized and rather specific for bone outcomes, but not for global health effects (4, 6). Low vitamin D levels are common in healthy newborns (33, 62) and are independently associated with various factors (skin color, diet, maternal levels and intake, supplements, and seasonality) and strengthen the controversy on the benefits of providing vitamin D integration during infancy.

## Current Scenario

The current reviews (54, 56–59) conclude that further studies are needed to evaluate the association between allergy and vitamin D. Vitamin D supplementation controlled studies aimed to clarify its role in food allergy development are still lacking.

Currently, there are more evidence to support vitamin D supplementation in pregnancy and infancy, in light of its positive effects. In fact, VDD may boost the risk of food allergy and sensitization among people with particular genotypes (32). Nevertheless, when studied alone, VDD was not associated with food allergy whereas, on the other hand, it was significantly related with specific gene polymorphisms, supplying evidence on food allergy. Also, the role of vitamin D beyond bone and calcium metabolism is not fully understood.

## Conclusions

The role of vitamin D beyond bone and calcium metabolism is alluring but not fully understood. The association between vitamin D and development of food allergy is contradictory (Tables 1, 2).

Potential relationships come from ecologic studies that associate lower sunlight exposure to food allergies. On the other hand, further research found that higher levels of vitamin D might raise the probability of allergic sensitization and food allergy. However, in light of a large literature linking the vitamin D levels to the onset of eczema and allergic diseases, this hormone must be considered as a further chance in the comprehension and treatment of atopic diseases. For this reason, there is an urgent need for well-planned randomized controlled trials on vitamin D supplementation, with particular regard to food allergy, to demonstrate that vitamin D might actually contribute to the prevention of allergic diseases.

## Author Contributions

GR, AG, and LB: conceptualization. AG, LB, and JC: resources. GR, AG, and RM: methodology and writing—review and editing. AG, LB, JC, MG, RM, and GR: writing—original draft preparation. RM and GR: supervision. All authors contributed to the article and approved the submitted version.

## Conflict of Interest

The authors declare that the research was conducted in the absence of any commercial or financial relationships that could be construed as a potential conflict of interest.
